# Data gaps towards health development goals, 47 low- and middle-income countries

**DOI:** 10.2471/BLT.21.286254

**Published:** 2021-11-04

**Authors:** Luhua Zhao, Bochen Cao, Elaine Borghi, Somnath Chatterji, Sebastian Garcia-Saiso, Arash Rashidian, Henry Victor Doctor, Marcelo D'Agostino, Humphrey C Karamagi, David Novillo-Ortiz, Mark Landry, Ahmad Reza Hosseinpoor, Abdisalan Noor, Leanne Riley, Adrienne Cox, Jun Gao, Steve Litavecz, Samira Asma

**Affiliations:** a Data, Analytics and Delivery for Impact Division, World Health Organization, Avenue Appia 20, 1211 Geneva 27, Switzerland.; b Regional Office for the Americas, World Health Organization, Washington, DC, United States of America (USA).; c Regional Office for the Eastern Mediterranean, World Health Organization, Cairo, Egypt.; d Regional Office for Africa, World Health Organization, Brazzaville, Congo.; e Regional Office for Europe, World Health Organization, Copenhagen, Denmark.; f Regional Office for South-East Asia, World Health Organization, New Delhi, India.; g Regional Office for the Western Pacific, World Health Organization, Manila, Philippines.; h RTI International, Research Triangle Park, USA.

## Abstract

**Objective:**

To assess the availability and gaps in data for measuring progress towards health-related sustainable development goals and other targets in selected low- and middle-income countries.

**Methods:**

We used 14 international population surveys to evaluate the health data systems in the 47 least developed countries over the years 2015–2020. We reviewed the survey instruments to determine whether they contained tools that could be used to measure 46 health-related indicators defined by the World Health Organization. We recorded the number of countries with data available on the indicators from these surveys.

**Findings:**

Twenty-seven indicators were measurable by the surveys we identified. The two health emergency indicators were not measurable by current surveys. The percentage of countries that used surveys to collect data over 2015–2020 were lowest for tuberculosis (2/47; 4.3%), hepatitis B (3/47; 6.4%), human immunodeficiency virus (11/47; 23.4%), child development status and child abuse (both 13/47; 27.7%), compared with safe drinking water (37/47; 78.7%) and births attended by skilled health personnel (36/47; 76.6%). Nineteen countries collected data on 21 or more indicators over 2015–2020 while nine collected data on no indicators; over 2018–2020 these numbers reduced to six and 20, respectively.

**Conclusion:**

Examining selected international surveys provided a quick summary of health data available in the 47 least developed countries. We found major gaps in health data due to long survey cycles and lack of appropriate survey instruments. Novel indicators and survey instruments would be needed to track the fast-changing situation of health emergencies.

## Résumé

**Objectif:**

Évaluer les données disponibles et les lacunes dans la mesure des progrès effectués pour atteindre les objectifs de développement durable liés à la santé et d'autres objectifs dans une série de pays à faible et moyen revenu.

**Méthodes:**

Nous nous sommes basés sur 14 enquêtes internationales menées au sein de la population afin d'analyser les systèmes de données sur la santé dans 47 pays figurant parmi les moins développés entre 2015 et 2020. Nous avons également passé en revue le matériel d'enquête pour déterminer s'il contenait des outils pouvant servir à mesurer 46 indicateurs sanitaires, tels que définis par l'Organisation mondiale de la Santé. Enfin, nous avons recensé le nombre de pays possédant des données sur les indicateurs issus de ces enquêtes.

**Résultats:**

Nous avons identifié des enquêtes dans lesquelles 27 indicateurs étaient mesurables. Les deux indicateurs relatifs aux urgences sanitaires étaient impossibles à évaluer à l'aide des enquêtes existantes. Les pourcentages les plus bas de pays recourant à des enquêtes pour récolter des données entre 2015 et 2020 s'appliquaient à la tuberculose (2/47; 4,3%), à l'hépatite B (3/47; 6,4%), au virus de l'immunodéficience humaine (11/47; 23,4%), au stade de développement des enfants et à leur maltraitance (tous deux 13/47; 27,7%). À l'autre extrémité se trouvaient l'accès à l'eau potable (37/47; 78,7%) et les accouchements assistés par du personnel qualifié (36/47; 76,6%). Dix-neuf pays ont recueilli des données sur minimum 21 indicateurs durant la période comprise entre 2015 et 2020, tandis que neuf n'en ont collecté sur aucun d'entre eux; entre 2018 et 2020, ces chiffres sont passés à 6 et 20 respectivement.

**Conclusion:**

L'examen d'une sélection d'enquêtes internationales nous a fourni un bref aperçu des données disponibles dans les 47 pays les moins développés. Nous avons décelé des lacunes considérables dans les données liées à la santé, en raison des longs cycles d'enquête et du manque de matériel adéquat. De nouveaux outils et indicateurs seraient nécessaires pour suivre l'évolution rapide de la situation en cas d'urgence sanitaire.

## Resumen

**Objetivo:**

Evaluar la disponibilidad y las carencias de datos para medir el progreso hacia los objetivos de desarrollo sostenible relacionados con la salud y otras metas en países seleccionados de ingresos bajos y medios.

**Métodos:**

Se utilizaron 14 encuestas internacionales de población para evaluar los sistemas de datos sanitarios en 47 países menos desarrollados durante los años 2015 a 2020. Se revisaron los instrumentos de las encuestas para determinar si incluían herramientas que se pudieran aplicar para medir 46 indicadores de salud que establece la Organización Mundial de la Salud. Se registró el número de países con datos disponibles sobre los indicadores de estas encuestas.

**Resultados:**

Las encuestas que se seleccionaron permitieron medir 27 indicadores, pero no fue posible medir los dos indicadores de emergencias sanitarias mediante las encuestas actuales. El porcentaje de países que aplicaron encuestas para recopilar datos entre 2015 y 2020 fue el más bajo para la tuberculosis (2/47; 4,3 %), la hepatitis B (3/47; 6,4 %), el virus de la inmunodeficiencia humana (11/47; 23,4 %), el estado de desarrollo infantil y el maltrato infantil (ambos 13/47; 27,7 %), en comparación con el agua potable (37/47; 78,7 %) y los partos a cargo de personal sanitario cualificado (36/47; 76,6 %). Diecinueve países recopilaron datos sobre 21 o más indicadores entre 2015 y 2020, mientras que 9 no recopilaron datos sobre ningún indicador; entre 2018 y 2020 estas cifras disminuyeron a 6 y 20, respectivamente.

**Conclusión:**

El análisis de encuestas internacionales seleccionadas proporcionó un breve resumen de los datos sanitarios disponibles en los 47 países menos desarrollados. Se encontraron importantes carencias en los datos sanitarios debido a los largos ciclos de las encuestas y a la falta de instrumentos de encuesta adecuados. Se necesitarían nuevos indicadores e instrumentos de encuesta para hacer un seguimiento de las situaciones de emergencia sanitaria, que cambian muy rápido.

## ملخص

الغرض تقييم مدى توافر البيانات، والثغرات الموجودة بها، في سبيل قياس التقدم نحو تحقيق أهداف التنمية المستدامة ذات الصلة بالصحة، والأهداف الأخرى في دول مختارة ذات دخل منخفض ودخل متوسط.

الطريقة استخدمنا 14 مسحًا دوليًا للسكان لتقييم أنظمة البيانات الصحية في أقل 47 دولة من حيث النمو خلال الأعوام من 2015 إلى 2020. وقمنا بمراجعة أدوات المسح لتحديد ما إذا كانت تحتوي على أدوات يمكن استخدامها لقياس 46 مؤشرًا من مؤشرات الصحة، قامت منظمة الصحة العالمية بتحديدها. لقد سجلنا عدد الدول التي لديها بيانات متوفرة عن المؤشرات من هذه المسوح.

النتائج كان سبعة وعشرون مؤشرا قابلا للقياس بواسطة المسوح التي حددناها. لم يكن مؤشرا الطوارئ الصحية قابلين للقياس بواسطة المسوحات الحالية. كانت النسبة المئوية للدول التي استخدمت المسوح لجمع البيانات خلال الفترة من 2015 إلى 2020 هي الأدنى بالنسبة لمرض السل (2/47؛ 4.3%)، والالتهاب الكبدي ب (3/47؛ 6.4%)، وفيروس نقص المناعة البشرية (11/47؛ 23.4%)، وحالة نمو الطفل وإساءة معاملة الأطفال (كلاهما 13/47؛ 27.7%)، مقارنة بمياه الشرب الآمنة (37/47؛ 78.7%)، وعمليات الولادة بواسطة أخصائيين صحيين مهرة (36/47؛ 76.6%). جمعت 19 دولة بيانات عن 21 مؤشرًا أو أكثر خلال الفترة من 2015 إلى 2020، بينما جمعت 9 دول بيانات عن عدم وجود مؤشرات؛ وخلال الفترة من 2018 إلى 2020 انخفضت هذه الأرقام إلى 6 و20 على التوالي.

الاستنتاج أدى فحص المسوحات الدولية المختارة إلى تقديم ملخص سريع للبيانات الصحية المتوفرة في الـ 47 دولة الأقل من حيث النمو. ووجدنا فجوات واسعة في البيانات الصحية بسبب دورات المسح الطويلة، ونقص أدوات المسح المناسبة. ستكون هناك حاجة إلى مؤشرات وأدوات مسح جديدة لتتبع الموقف سريع التغير للطوارئ الصحية.

## 摘要

**目的:**

评估在特定中低收入国家可用于衡量与卫生相关的可持续发展目标和其他目标进展的数据可得性及空缺。

**方法:**

我们使用 14 项国际人口调查评估了 47 个最不发达国家 2015 年至 2020 年间的卫生数据系统。我们审阅了其调查问卷和其他调查方式以评估这些人口调查是否可用于衡量世界卫生组织制定的 46 个与卫生相关的指标。我们记录了通过上述调查来获取卫生数据以衡量这些指标的国家数量。

**结论:**

二十七项指标可通过我们选定的调查衡量。两项与突发卫生事件相关的指标不能被当前调查所衡量。对比安全饮用水 (37/47；78.7%) 和由专业医护人员接生的出生率 (36/47；76.6%)，2015 年至 2020 年间使用调查收集以下方面数据的国家比例最低：结核病 (2/47；4.3%)、乙型肝炎 (3/47；6.4%)、人类免疫缺陷病毒 (11/47；23.4%)、儿童发展状况和虐待儿童(均为 13/47；27.7%)。2015 年至 2020 年间十九个国家收集了 21 项或更多指标的数据，而九个国家未收集任何指标的数据，上述数值在 2018 年至 2020 年间分别减至六和 20。

**结论:**

通过审阅特定国际人口调查可提供对 47 个最不发达国家可用卫生数据的快捷评估。造成这些国家卫生数据空缺的主要原因是较长的调查周期以及缺乏适当的调查手段。需要新的指标和调查工具以追踪快速变化的突发卫生事件。

## Резюме

**Цель:**

Оценить наличие необходимых данных и пробелы в них для измерения прогресса в достижении связанных со здоровьем целей в области устойчивого развития и других задач в отдельных странах с низким и средним уровнем доходов.

**Методы:**

Авторы применили 14 международных обследований населения для оценки систем данных о состоянии здоровья населения в 47 наименее развитых странах за 2015–2020 годы. Авторы изучили средства обследований, чтобы определить, содержат ли они инструменты, которые можно использовать для измерения 46 показателей, связанных с состоянием здоровья, определенных Всемирной организацией здравоохранения. Авторы зарегистрировали количество стран, по которым доступны данные по показателям этих обследований.

**Результаты:**

Двадцать семь показателей можно было измерить с помощью выбранных обследований. Два показателя чрезвычайных ситуаций в области здравоохранения не поддавались количественной оценке с помощью текущих обследований. Процент стран, которые использовали обследования для сбора данных за 2015–2020 годы, был самым низким по туберкулезу (2/47; 4,3%), гепатиту B (3/47; 6,4%), вирусу иммунодефицита человека (11/47; 23,4%), статусу развития ребенка и жестокого обращения с детьми (13/47; 27,7%) по сравнению с безопасной питьевой водой (37/47; 78,7%) и родами, проводимыми квалифицированным медицинским персоналом (36/47; 76,6%). Девятнадцать стран собрали данные по 21 или более показателям за 2015–2020 годы, в то время как девять стран не собрали данные ни по каким показателям; за 2018–2020 годы их количество сократилось до шести и 20 соответственно.

**Вывод:**

Изучение отдельных международных обследований позволило получить краткую характеристику данных о состоянии здоровья в 47 наименее развитых странах. Авторы обнаружили серьезные пробелы в данных о состоянии здоровья из-за длительных циклов обследований и отсутствия соответствующих инструментов обследований. Для отслеживания быстро меняющейся ситуации чрезвычайных ситуаций в области здравоохранения потребуются новые показатели и инструменты обследования.

## Introduction

The United Nations (UN) *Transforming our world: the 2030 agenda for sustainable development* is a global plan shared by Member States for a far healthier, more prosperous world.^1^^,^^2^ Fifty-seven of the 232 UN sustainable development goal (SDG) indicators were subsequently identified as health-related indicators by the World Health Organization (WHO) in 2019 with two more added afterwards.^3^^,^^4^ In alignment with the SDGs, WHO Member States approved the *Thirteenth general programme of work 2019−2023* in 2018 and set the triple billion targets to accelerate delivering impact in countries.^5^ The results framework of the general programme of work identified 46 health outcome indicators to track countries’ progress towards the triple billion targets,^6^ including 39 SDG indicators and seven non-SDG indicators.^7^ The seven non-SDG indicators were approved in World Health Assembly (WHA) resolutions including two that relate to health emergencies. These 46 indicators assist WHO Member States to stay focused and accelerate their progress towards achieving the SDGs and the triple billion targets.

The recommendations of the thirteenth general programme of work would require gathering data in many dimensions, yet little is known about the efforts needed by countries to measure these 46 indicators. Researchers have estimated that 12 data systems would be required in each country to monitor 57 health-related SDG indicators.^3^ A similar number of systems would likely be needed to measure the 46 indicators. Representative household surveys, civil registration and vital statistics, and other administrative health data systems are the primary and preferred data source for monitoring health indicators.^8^ Regular population-based health surveys are a key component of well-functioning data systems for surveying populations and health risks.^9^ Household surveys can provide data for an estimated 29 of the 57 health-related indicators.^3^ Additionally, valid measurement of some indicators, such as out-of-pocket health expenditure, are mostly achieved through surveys. Many countries, however, especially low- and middle-income countries, lack the technical capacity and financial resources to perform these surveys, and have relied on development partners to implement surveys.^10^

The WHO SCORE (survey, count, optimize, review, enable) global report in 2020 revealed that most low-income countries relied heavily on external funding for surveys, with only 3% of the surveys fully funded by a government.^9^ International surveys provided essential health data and this was sometimes the only data available in these countries to monitor some important health trends. Reviewing the survey methods and procedures in low- and middle-income countries would offer valuable insight into whether and to what extent key health data, measurable by the 46 indicators, were available in these countries. Our literature search showed that not much work has been done in this area. The UN Intersecretariat Working Group on Household Surveys conducted a review to map the SDG global indicators to household surveys.^11^ The report found that 77 of the SDG indicators could be sourced from household surveys; however, the availability of the data was not assessed. 

We therefore aimed to assess the availability and gaps in the relevant data and to explore new methods for improving health data in low- and middle-income countries. Our study focused on reviewing international surveys that have been implemented in these countries. We reviewed each survey to determine if it could measure any of the 46 health outcome indicators defined by the WHO. We also reviewed the implementation of the surveys between the years 2015 and 2020 in 47 low- and middle-income countries that are designated by the UN as the least developed countries.^12^ These countries lack adequate local capacity, and depend heavily on surveys sponsored by international partners to provide essential health data. We therefore found it feasible to assess their health data systems through examining the international surveys used. The results provide evidence on countries’ progress towards the health-related SDGs. We also propose exploring new methods to reduce the data gaps through surveys, particularly for timely and rapid data collection in health emergency settings.

## Methods

We identified potential international population surveys based on a literature review and consultation with experts across WHO technical programmes. We included surveys in our study if they covered health topics; were coordinated or facilitated by international agencies or partners; had been implemented in multiple least developed countries since 2000 using nationally representative samples; and had a core questionnaire or instrument and a standard protocol. We included surveys concerning human immunodeficiency virus (HIV), tuberculosis, malaria and hepatitis B only if rapid diagnosis tests or laboratory testing were used in the surveys in addition to data collected through oral interviews.

For each type of survey, we conducted an extensive online review to identify standard and country-specific survey instruments. We examined the instruments to determine whether they contained tools (such as survey questions, health examinations or collection of biomarkers) that could potentially be used to measure any of the 46 health outcome indicators. When the country-specific survey instrument was not available, we used the standard international questionnaire for assessment. We categorized each indicator into one of three groups according to its measurability: (i) measurable (if the surveys we studied contained suitable questions, health examinations or biomarkers); (ii) potentially measurable (if the surveys we studied did not contain suitable questions, health examinations or biomarkers but such tools could be formulated and added); and (iii) not measurable (if suitable questions, health examinations or biomarkers could not be formulated for any surveys). If surveys containing tools to measure an indicator were conducted in a country between 2015 and 2020, we assumed that data were available for this indicator in this country for this period. We judged that an indicator was not measurable by surveys if its calculation involved important information unavailable from surveys. For SDG 3.4 (incidence of HIV, tuberculosis, malaria and hepatitis B infections), for example, we judged that surveys needed to include rapid diagnostic tests or laboratory testing to measure the indicators.

For all 47 least developed countries^12^ we assessed the availability of data for all indicators that were deemed measurable by the surveys we studied. We did this by mapping the indicators each survey can measure. We first assessed data availability and the number of indicators measured by the surveys for the 47 countries for the period of 2015–2020. We conducted a second analysis using the period 2018–2020 to evaluate the impact of survey frequency on availability of health data in these countries. For each indicator, we recorded the number of countries that could measure it and then calculated the percentage out of the 47 countries. 

## Results

We identified 14 different surveys that could measure at least one of the 46 health outcome indicators from 2015 to 2020 (Table 1). The surveys included comprehensive multi-topic surveys such as demographic and health surveys (DHS), labour force surveys, living standards measurement surveys and multiple indicator cluster surveys (MICS), as well as single-topic surveys such as household income and expenditure survey and reproductive health surveys. More details of these surveys are provided in the authors’ data repository.^13^

**Table 1:** Suitability of surveys and availability of data for measuring 46 WHO health outcome indicators in 47 low- and middle-income countries, 2015–2020

Health indicator^a^	Suitability for measurement by available surveys^b^	Survey used to measure indicator	No. (%) of countries with data available on indicator (*n* = 47)^c^	
			Years 2015–2020	Years 2018–2020
SDG 1.5.1: Number of deaths, missing persons and directly affected persons attributed to disasters per 100 000 population	Not measurable	NA	NA	NA
SDG 1.a.2: Proportion of total government spending on essential services (education, health and social protection)	Not measurable	NA	NA	NA
SDG 2.2.1: Prevalence of stunting (height for age < −2 standard deviations from the median of the WHO child growth standards) among children under 5 years of age	Measurable	DHS; living standards measurement survey; MICS	35 (74.5)	23 (48.9)
SDG 2.2.2: Prevalence of malnutrition (weight for height > +2 or < −2 standard deviations from the median of the WHO child growth standards) among children under 5 years of age, by type (wasting)	Measurable	DHS; living standards measurement survey; MICS	35 (74.5)	23 (48.9)
SDG 2.2.2: Prevalence of malnutrition (weight for height > +2 or < −2 standard deviations from the median of the WHO child growth standards) among children under 5 years of age, by type (overweight)	Measurable	DHS; living standards measurement survey; MICS	35 (74.5)	23 (48.9)
SDG 3.1.1: Maternal mortality ratio	Measurable	DHS	25 (53.2)	12 (25.5)
SDG 3.1.2: Proportion of births attended by skilled health personnel	Measurable	AIDS indicator surveys; DHS; household income and expenditure survey; labour force survey; living standards measurement survey; MICS; reproductive health survey	36 (76.6)	24 (51.1)
SDG 3.2.1: Under-five mortality rate	Measurable	DHS; living standards measurement survey; MICS; reproductive health survey	35 (74.5)	23 (48.9)
SDG 3.2.2: Neonatal mortality rate	Measurable	DHS; living standards measurement survey; MICS; reproductive health survey	35 (74.5)	23 (48.9)
SDG 3.3.1: Number of new HIV infections per 1000 uninfected population, by sex, age and key populations	Measurable	DHS; AIDS indicator survey	11 (23.4)	4 (8.5)
SDG 3.3.2: Tuberculosis incidence per 100 000 population	Measurable	Tuberculosis prevalence survey	2 (4.3)	0 (0.0)
SDG 3.3.3: Malaria incidence per 1000 population	Measurable	DHS; malaria indicator survey	18 (38.3)	8 (17.0)
SDG 3.3.4 Hepatitis B incidence per 100 000 population	Measurable	DHS	3 (6.4)	3 (6.4)
SDG 3.3.5 Number of people requiring interventions against neglected tropical diseases	Potentially measurable	NA	NA	NA
SDG 3.4.1: Mortality rate attributed to cardiovascular disease, cancer, diabetes or chronic respiratory disease	Potentially measurable	NA	NA	NA
SDG 3.4.2: Suicide mortality rate	Measurable	DHS; world mental health survey	25 (53.2)	12 (25.5)
SDG 3.5.1: Coverage of treatment interventions (pharmacological, psychosocial and rehabilitation and aftercare services) for substance use disorders	Potentially measurable	NA	NA	NA
SDG 3.5.2: Harmful use of alcohol, defined according to the national context as alcohol per capita consumption (aged 15 years and older) within a calendar year in litres of pure alcohol	Measurable	MICS; STEPwise approach to surveillance; world mental health survey	24 (51.1)	13 (27.7)
SDG 3.6.1: Death rate due to road traffic injuries	Measurable	DHS	25 (53.2)	12 (25.5)
SDG 3.7.1: Proportion of women of reproductive age (aged 15–49 years) who have their need for family planning satisfied with modern methods	Measurable	DHS; MICS; performance monitoring for action; reproductive health survey	35 (74.5)	25 (53.2)
SDG 3.8.1: Coverage of essential health services (defined as the average coverage of essential services based on tracer interventions that include reproductive, maternal, newborn and child health, infectious diseases, noncommunicable diseases and service capacity and access, among the general and the most disadvantaged population)	Not measurable	NA	NA	NA
SDG 3.8.2: Proportion of population with large household expenditures on health as a share of total household expenditure or income	Measurable	Household income and expenditure survey; labour force survey; living standards measurement survey; reproductive health survey; world mental health survey; world health survey	16 (34.0)	3 (6.4)
SDG 3.9.1: Mortality rate attributed to household and ambient air pollution	Not measurable	NA	NA	NA
SDG 3.9.2: Mortality rate attributed to unsafe water, unsafe sanitation and lack of hygiene (exposure to unsafe Water, Sanitation and Hygiene for All (WASH) services)	Potentially measurable	NA	NA	NA
SDG 3.9.3: Mortality rate attributed to unintentional poisoning	Potentially measurable	NA	NA	NA
SDG 7.1.2: Proportion of population with primary reliance on clean fuels and technology	Measurable	AIDS indicator surveys; DHS; household income and expenditure survey; labour force survey; living standards measurement survey; tuberculosis prevalence survey; reproductive health survey	27 (57.4)	13 (27.7)
SDG 11.6.2: Annual mean level of fine particulate matter (such as PM_2.5_ and PM_10_) in cities (population weighted)	Not measurable	NA	NA	NA
SDG 3.a.1: Age-standardized prevalence of current tobacco use among persons aged 15 years and older	Measurable	DHS; global adult tobacco survey; living standards measurement survey; MICS; STEPwise approach to surveillance	36 (76.6)	24 (51.1)
SDG 3.b.1: Proportion of the target population covered by all vaccines included in their national programme	Measurable	DHS	25 (53.2)	12 (25.5)
SDG 3.b.3: Proportion of health facilities that have a core set of relevant essential medicines available and affordable on a sustainable basis	Not measurable	NA	NA	NA
SDG 3.c.1: Health worker density and distribution	Not measurable	NA	NA	NA
SDG 3.d.1: International Health Regulations capacity and health emergency preparedness	Not measurable	NA	NA	NA
SDG 4.2.1: Proportion of children under 5 years of age who are developmentally on track in health, learning and psychosocial well-being, by sex	Measurable	MICS (2018 and after)	13 (27.7)	13 (27.7)
SDG 5.2.1: Proportion of ever-partnered women and girls aged 15 years and older subjected to physical, sexual or psychological violence by a current or former intimate partner in the previous 12 months, by form of violence and by age	Measurable	DHS; MICS	34 (72.3)	23 (48.9)
SDG 5.6.1: Proportion of women aged 15–49 years who make their own informed decisions regarding sexual relations, contraceptive use and reproductive health care	Measurable	DHS; performance monitoring for action	27 (57.4)	15 (31.9)
SDG 6.1.1: Proportion of population using safely managed drinking-water services	Measurable	AIDS indicator survey; DHS; household income and expenditure survey; labour force survey; living standards measurement survey; MICS; malaria indicator survey; tuberculosis prevalence survey; reproductive health survey	37 (78.7)	26 (55.3)
SDG 6.2.1: Proportion of population using safely managed sanitation services, including a hand-washing facility with soap and water	Measurable	DHS; household income and expenditure survey; labour force survey; living standards measurement survey; MICS; malaria indicator survey; tuberculosis prevalence survey; reproductive health survey	37 (78.7)	26 (55.3)
SDG 16.2.1: Proportion of children aged 1–17 years who experienced any physical punishment and/or psychological aggression by caregivers in the past month	Measurable	MICS (2018 and after)	13 (27.7)	13 (27.7)
Health emergency indicator: Vaccine coverage of at-risk groups for epidemic or pandemic prone diseases	Not measurable	NA	NA	NA
Health emergency indicator: Proportion of vulnerable people in fragile settings provided with essential health services	Not measurable	NA	NA	NA
WHA 68.3: Number of cases of poliomyelitis caused by wild poliovirus	Not measurable	NA	NA	NA
WHA 68.7: Patterns of antibiotic consumption at national level	Potentially measurable	NA	NA	NA
WHA 67.25, WHA 68.7: Percentage of bloodstream infections due to antimicrobial resistant organisms	Not measurable	NA	NA	NA
WHA 66.10: Age-standardized prevalence of raised blood pressure among persons aged 18+ years (defined as systolic blood pressure of ≥ 140 mmHg and/or diastolic blood pressure ≥ 90 mmHg)	Measurable	DHS; STEPwise approach to surveillance	27 (57.4)	13 (27.7)
WHA 66.10: Percentage of people protected by effective regulation on trans-fats	Not measurable	NA	NA	NA
WHA 66.10: Prevalence of obesity	Measurable	DHS; STEPwise approach to surveillance	27 (57.4)	13 (27.7)

AIDS: acquired immunodeficiency syndrome; DHS: demographic and health survey; MICS: multiple indicator cluster survey; NA: not applicable; SDG: sustainable development goal; WHA: World Health Assembly; WHO: World Health Organization.

^a^ The 46 health outcome indicators were those identified in WHO’s thirteenth general programme of work for measuring the UN sustainable development goals and triple billion targets.^6^

^b^ We classified indicators as: measurable, if the surveys we studied contained suitable questions, health examinations or biomarkers; potentially measurable, if the surveys we studied did not contain suitable questions, health examinations or biomarkers but such tools could be formulated and added; or not measurable, if suitable questions, health examinations or biomarkers could not be formulated for any surveys.

^c^ The 47 low- and middle-income countries are: Afghanistan, Angola, Bangladesh, Benin, Bhutan, Burkina Faso, Burundi, Cambodia, Central African Republic, Chad, Comoros, Democratic Republic of the Congo, Djibouti, Eritrea, Ethiopia, Gambia, Guinea, Guinea-Bissau, Haiti, Kiribati, Lao People's Democratic Republic, Lesotho, Liberia, Madagascar, Malawi, Mali, Mauritania, Mozambique, Myanmar, Nepal, Niger, Rwanda, Sao Tome and Principe, Senegal, Sierra Leone, Solomon Islands, Somalia, South Sudan, Sudan, Timor-Leste, Togo, Tuvalu, Uganda, United Republic of Tanzania, Vanuatu, Yemen, Zambia.

DHS were able to measure the greatest number of indicators (22 indicators), followed by MICS (14 indicators), living standards measurement surveys (11 indicators), reproductive health surveys (eight indicators), household income and expenditure surveys (five indicators) and labour force surveys (five indicators). There were major overlaps in survey contents among them. Nine indicators measured by DHS could also be measured by MICS and living standards measurement surveys. Out of 14 indicators measurable by MICS, 11 indicators could also be measured by DHS. Similarly, 10 of 11 indicators that are measured by living standards measurement surveys are also measured by DHS.

We found that 27 indicators were measurable by the selected surveys. The percentages of countries that used the surveys to measure each indicator between 2015 and 2020 are listed in Table 1. The percentages were lowest for tuberculosis incidence (SDG 3.3.2, two countries, 4.3%), hepatitis B incidence (SDG 3.3.4, three countries, 6.4%), new HIV infection (SDG 3.3.1, 11 countries, 23.4%), child development status and child abuse (SDG 4.2.1 and SDG 16.2.1, both 13 countries, 27.7%). Except for the indicators on households with large health expenditures (SDG 3.8.2, 16 countries, 34.0%) and malaria incidence (SDG 3.3.3, 18 countries, 38.3%), the remaining indicators all achieved coverage of 50% or more. The highest coverage was for safe drinking-water and sanitation services (SDG 6.1.1 and SDG 6.2.1),which both reached 78.7% (37 countries), followed by SDG 3.1.2 (birth attended by skilled health personnel) and SDG 3.a.1 (tobacco use) at 76.6% (36 countries). The percentage coverage was also high for SDG 2.2.1 (stunting), SDG 2.2.2 (overweight and wasting), SDG 3.2.1 (under-five mortality rate), SDG 3.2.2 (neonatal mortality rate) and SDG 3.7.1 (women satisfied with modern family planning methods), measured in 35 countries (74.5%) each. About half of the countries had data to measure adult obesity (WHA 66.10), hypertension (WHA 66.10) and primary reliance on clean fuel (SDG 7.1.2), all measured in 27 countries (57.4%). When the observation period changed to 2018–2020, the percentages dropped for all 27 indicators, with most countries decreasing their measurement of the indicators by 20 or more percentage points.

Thirteen indicators were not measurable by surveys. These indicators fell into six groups: (i) indicators that concern health facility and health workers, including SDG 3.b.3 (health facility operation) and SDG 3.c.1 (health workforce); (ii) indicators that involve government policies, including WHA 66.10 (trans-fats regulation), SDG 3.d.1 (International Health Regulations compliance) and SDG 1.a.2 (government spending); (iii) indicators that involve sophisticated testing and diagnosis, including WHA 68.3 (poliomyelitis) and WHA 67.25 (antimicrobial resistance); (iv) indicators that are usually reported through civil registration and vital statistics and administrative data systems, including SDG 1.5.1 (death from disasters) and SDG 11.6.2 (fine particulate matter); (v) indicators that involve complex calculation algorithms, including SDG 3.9.1 (mortality rate attributable to household and ambient environment); and (vi) composite indicators that involve multiple tracer variables, including SDG 3.8.1 (coverage of universal health care) and the two indicators that are related to readiness and response to health emergencies.

The remaining six indicators were potentially measurable by surveys, but were not measured by the surveys we studied. These indicators were SDG 3.3.5 (intervention against neglected tropical diseases), SDG 3.5.1 (treatment intervention for substance abuse disorder), WHA 68.7 (antibiotic consumption level) and SDG 3.4.1, SDG 3.9.2 and SDG 3.9.3 (mortality indicators).

Our results also provided information to assess how the 47 countries fared overall in tracking public health trends. Over the period 2015–2020, 19 of the countries collected data on 21 or more of the 46 health outcome indicators and 16 countries had data on 11 to 20 indicators; nine countries collected no data, of which five countries were from WHO African Region, three from Western Pacific Region and one from Eastern Mediterranean Region (Table 2). When we analysed the time period 2018–2020, the number of countries with data on 21 or more indicators dropped to six, and countries with no data increased to 20, across five WHO regions.

**Table 2:** Numbers of WHO health outcome indicators measured by international surveys in 47 low- and middle-income countries, 2015–2020

Country by WHO Region	No. of indicators measured (maximum 46)	
	Years 2015–2020	Years 2018–2020
**African Region**		
Angola	21	0
Benin	21	20
Burkina Faso	5	5
Burundi	20	0
Central African Republic	14	14
Chad	22	14
Comoros	0	0
Democratic Republic of the Congo	15	15
Djibouti	0	0
Eritrea	0	0
Ethiopia	22	19
Gambia	24	23
Guinea	20	20
Guinea-Bissau	14	14
Lesotho	14	14
Liberia	22	20
Madagascar	24	14
Malawi	25	14
Mali	22	21
Mauritania	22	21
Mozambique	14	6
Niger	22	0
Rwanda	22	21
Sao Tome and Principe	14	14
Senegal	23	21
Sierra Leone	22	20
Somalia	0	0
South Sudan	0	0
Sudan	4	0
Uganda	22	5
United Republic of Tanzania	21	1
Zambia	21	20
**Americas Region**		
Haiti	20	0
**Eastern Mediterranean Region**		
Afghanistan	19	0
Yemen	0	0
**South-East Asia Region**		
Bangladesh	24	22
Bhutan	5	0
Myanmar	20	0
Nepal	23	16
Timor-Leste	19	0
**Western Pacific Region**		
Cambodia	0	0
Kiribati	16	14
Lao People's Democratic Republic	12	0
Solomon Islands	0	0
Togo	13	0
Tuvalu	14	14
Vanuatu	0	0

WHO: World Health Organization.

Notes: The 46 health outcome indicators were those identified in WHO’s thirteenth general programme of work for measuring the UN sustainable development goals and triple billion targets.^6^ Detailed information at country level is available online from the authors’ data repository.^14^

## Discussion

As WHO Member States are mandated to track and report their progress at country level towards SDGs and triple billion targets,^5^ it is essential that they collect and use health data to identify the key priority areas for improvement. Well-functioning health information systems, particularly civil registration and vital statistics and other administrative data systems, were lacking in the 47 least developed countries.^12^ WHO’s SCORE global report estimated that 40% of annual deaths went unregistered globally, particularly in Africa.^9^ Many low- and middle-income countries, particularly the least developed countries, relied on international surveys as the primary source for many essential health data. We were therefore able to assess the key health data gaps in the studied countries by examining international surveys. In conjunction with the 46 health outcome indicators identified by WHO, international surveys can provide a relatively simple and quick way to assess gaps in essential health data in these countries.

We found data gaps in the 47 countries studied. None of the countries had data for all 27 indicators measurable by surveys between 2015 and 2020. The most monitored indicators were measured in about three quarters of countries. The least tracked indicators were those for infectious diseases, particularly tuberculosis and hepatitis B, each of which were reported by 4.3% and 6.4% of the countries, respectively. This result is expected given that the diagnosis of these health conditions requires laboratory work that was not provided by most surveys. Furthermore, less than one third of the countries had data to monitor child development status and child abuse, a sign that more investment is needed to track children’s welfare.

The pandemic of coronavirus disease 2019 (COVID-19) has highlighted the urgency of timely and accurate measurement of population mortality, as well as the need for rapid collection of key data for informing policies and actions. Household surveys are one of the major sources for mortality data in countries that do not have reliable civil registration and vital statistics systems. However, surveys often lack instruments to obtain mortality statistics. Five of the 10 mortality indicators identified by the WHO thirteenth general programme of work are either not measurable by surveys or potentially measurable but not measured by the surveys we studied. Sibling survival history – a method commonly used in surveys to measure mortality – only provides all-cause and pregnancy-related mortality, and tends to produce sparse data among older adults. Researchers have tested additional questions for sibling survival history to determine HIV/AIDS mortality,^15^ while others have explored the idea of a social network survival method to capture death data more efficiently.^16^ Further research is needed to develop more innovative methods to enhance the capacity of surveys for rapid measurement of population mortality.

The COVID-19 pandemic also showed the need to rapidly assess the local situation during health emergencies. Health emergency readiness is usually measured through reports based on the International Health Regulations,^17^^,^^18^ which relies on government reporting prepared in advance and does not provide up-to-date information. Surveys can be used to obtain quick results. However, out of the 46 indicators studied, only two indicators were directly related to health emergencies and neither were measurable directly by surveys. Developing suitable indicators for population-based surveys is needed to track and monitor the rapidly changing situation during health emergencies.

Heavy reliance on a few surveys can have negative consequences. Comprehensive population surveys such as DHS and MICS take significant time, manpower and financial investment to implement, and are thus usually conducted every 5 years or more.^11^ Countries relying on these surveys may find themselves without data to monitor important health trends between survey cycles, which can be affected by unexpected events such as pandemics. In the current study, the number of countries with no data for the studied indicators rose from nine to 20 when the time period studied was shortened from 2015–2020 to 2018–2020, showing the risk of dependence on a small number of surveys. Our analyses revealed that the difference was primarily due to the scheduling of DHS and MICS. Greater efforts are needed to find new ways of filling the data gaps between these surveys.

Moreover, most health surveys still rely on face-to-face interviews for data collection. As the COVID-19 pandemic has shown, this method is often not feasible due to safety concerns and travel restrictions imposed by authorities. Many countries have responded by adopting mobile phone surveys and web-based surveys as alternatives,^19^ even though guidelines have been developed to restore the capacity to run in-person interviews. Adapting to the challenges posed by COVID-19 would help not only to collect data that is urgently needed for responding to the pandemic but also to prepare surveys for other emergencies and rapid responses. Mobile phone surveys are suitable for this purpose as they can collect data rapidly with lower costs. Such surveys can also be used to quickly fill the specific data gaps in combination with prior health data evaluations, such as the assessment introduced in our study and WHO SCORE technical package.^9^^,^^20^ International regulations need to be developed to reduce unnecessary ethical review requirements for non-sensitive surveys and minimize restrictions on mobile network access. Such barriers can offset the speediness of mobile phone surveys.

Data that are comparable across countries and consistent over time are important not only for national planning and evaluation of health systems but also for global assessment and progress tracking. Previous studies have indicated poor correlation of data on SDG indicators across various reports due to differences in indicator definitions, data sources, data processing and methods of synthesis.^21^^,^^22^ International surveys may adopt indicator definitions that are not comparable with those approved by WHO. For example, DHS conduct HIV testing, but only for anonymous respondents between 15 and 49 years old,^23^ whereas measuring progress on SDG 3.3.1 requires HIV indicators for broader populations disaggregated by sex, age group and other categories. This inconsistency would likely represent a challenge for tracking progress towards the SDG and triple billion targets in countries that rely on these data. Despite the efforts among international partners to coordinate the monitoring of health trends, greater collaboration is needed to increase data comparability and close data gaps.

This study has a few limitations. First, we did not include important data sources such as civil registration and vital statistics and administrative data sources. However, the impact on our findings should be minimal as most of the indicators examined rely on surveys as the primary data source, and civil registration and vital statistics systems are mostly too poorly developed in the 47 least developed countries to play a key role. Second, when details were not available online for some countries, we used core questionnaires from the same survey series for assessment. This approach could cause potential discrepancies for countries with tailored questionnaires. Last, low data quality can render some data unusable. However, as data quality varies across surveys and can be influenced by many factors that are difficult to measure, this issue, although important, was not included in the discussion.

Examining international surveys provided a quick summary of essential health data in the 47 least developed countries. Current population surveys, the bulk of data sources, are not frequent enough to provide timely monitoring for health-related indicators in these countries. Population surveys also lacked suitable instruments to collect data for informing actions during health emergencies. We propose exploring novel indicators and survey instruments to monitor and track health emergencies, along with rapid data collection methods such as mobile phone surveys.
